# Why do humans need thrombospondin-1?

**DOI:** 10.1007/s12079-023-00722-5

**Published:** 2023-01-23

**Authors:** Sukhbir Kaur, David D. Roberts

**Affiliations:** Laboratory of Pathology, Center for Cancer Research, National Cancer Institute, National Institutes of Health, Building 10 Room 2S235, 10 Center Dr, Bethesda, MD 20892-1500 USA

**Keywords:** Human genetic variation, Thrombospondin-1, Loss of function variants, Matricellular proteins, Essential genes

## Abstract

Matricellular proteins comprise several families of secreted proteins that function in higher animals at the interface between cells and their surrounding extracellular matrix. Targeted gene disruptions that result in loss of viability in mice have revealed critical roles for several matricellular proteins in murine embryonic development, including two members of the cellular communication network (CCN) gene family. In contrast, mice lacking single or multiple members of the thrombospondin (THBS) gene family remain viable and fertile. The frequency of loss of function mutants, identified using human deep exome sequencing data, provided evidence that some of the essential genes in mice, including *Ccn1*, are also essential genes in humans. However, a deficit in loss of function mutants in humans indicated that *THBS1* is also highly loss-intolerant. In addition to roles in embryonic development or adult reproduction, genes may be loss-intolerant in humans because their function is needed to survive environmental stresses that are encountered between birth and reproduction. Laboratory mice live in a protected environment that lacks the exposures to pathogens and injury that humans routinely face. However, subjecting *Thbs1*^*−/−*^ mice to defined stresses has provided valuable insights into functions of thrombospondin-1 that could account for the loss-intolerance of *THBS1* in humans.

**Graphical Abstract:**

**Figure:**
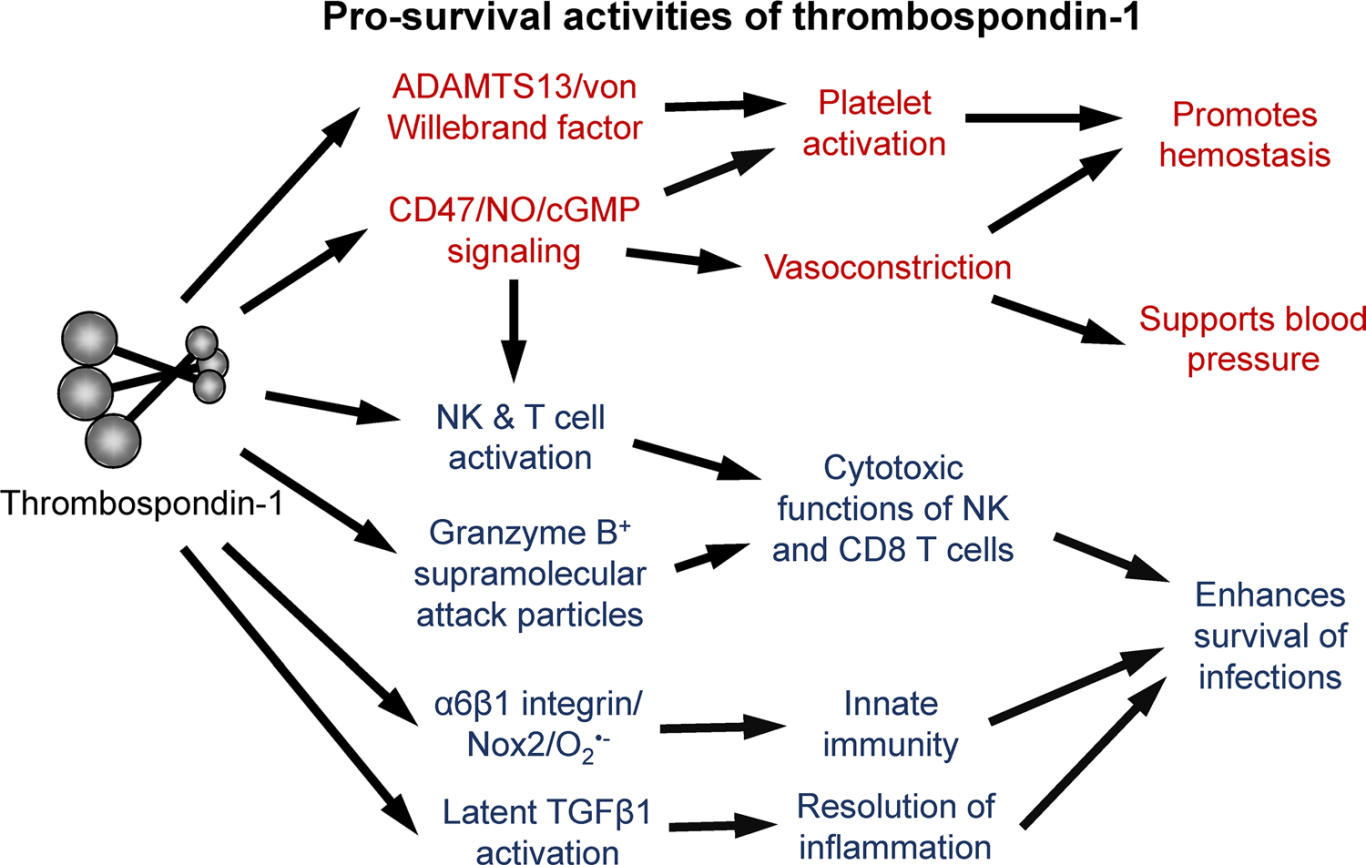
Stress response models using transgenic mice have identified protective functions of thrombospondin-1 in the cardiovascular system (red) and immune defenses (blue) that could account for its intolerance to loss of function mutants in humans

## Introduction

Several families of secreted proteins have been identified in higher animals that regulate cell behavior by interacting with structural components of the extracellular matrix, secreted cytokines and proteases, and specific cell surface receptors. These are collectively known as matricellular proteins (Bornstein 1995; Murphy-Ullrich and Sage 2014). Matricellular proteins include members of the thrombospondin, tenascin, secreted protein acidic and rich in cysteine (SPARC), small integrin-binding ligand N-linked glycoproteins (SIBLING), and cellular communication network (CCN) gene families (Murphy-Ullrich and Sage 2014; Leask 2020).

Mutations have been identified in matricellular protein genes including *CCN6*, *SPARC*, *SMOC1*, *SPOCK1*, *TNXB*, *DMP1*, *DSPP*, *THBS1*, *THBS2*, and *COMP* that cause inherited genetic disorders in humans or are linked to increased disease risk (Hurvitz et al. 1999; Topol et al. 2001; Bristow et al. 2005; Stenina et al. 2007; Burke et al. 2009; Abouzeid et al. 2011; Okada et al. 2011; Rainger et al. 2011; Staines et al. 2012; Dhamija et al. 2014; Mendoza-Londono et al. 2015; Posey et al. 2018). Insights into whether any matricellular protein genes are essential in mammals have been gained by studying targeted gene knockouts in mice. These studies demonstrated that *CCN1*, *CCN2*, and *SMOC1* are essential for viability (Mo et al. 2002; Ivkovic et al. 2003; Mo and Lau 2006; Okada et al. 2011). In contrast, the other homozygous null mice reported to date are viable and fertile (Jones and Jones 2000; Svensson et al. 2002; Hankenson et al. 2005a, 2005b; Kutz et al. 2005; Bradshaw 2009; Midwood and Orend 2009; Canalis et al. 2010; Bouleftour et al. 2016).

## Are essential matricellular genes in mice also essential in humans?

The role of specific genes in human viability cannot be directly addressed, but many recessive genes involved in lethal genetic diseases in humans impair viability when disrupted in mice. Conversely, loss of viability in mice may be predictive of an essential function in humans (Dawes et al. 2019). Two of the matricellular protein genes known to be essential for mouse viability are members of the CCN family (Mo et al. 2002; Ivkovic et al. 2003; Mo and Lau 2006). We recently examined the frequency of LoF mutations for CCN family members in The Genome Aggregation Database (gnomAD, v2.1.1), which includes 125,748 deep-sequenced exomes and 15,701 full genome sequences from unrelated individuals with no known severe pediatric genetic disease risk (Karczewski et al. 2020). Over our lifespan, germinal DNA is subject to random mutations caused by background ionizing radiation and oxidative stress. Although epigenetic modifications may partially protect some essential genes from such random mutations (Zhang 2022), the expected number of LoF mutations is proportional to the length of the open reading frame (Lek et al. 2016). Based on the length of the *CCN1* open reading frame, 17.2 LoF mutants were expected among the 141,456 individuals in gnomAD, but only 3 LoF mutants were observed. Therefore, *CCN1* had a significantly elevated probability of LoF intolerance (pLI = 0.71) (Kaur and Roberts 2021). This is consistent with the essential function of *Ccn1* in mouse prenatal development (Mo et al. 2002; Mo and Lau 2006) and establishes that mutation frequency data can be informative for evaluating loss-intolerance for matricellular protein genes in a human population.

Consistent with its essential function in mice (Ivkovic et al. 2003), the 7 observed *CCN2* LoF mutants in gnomAD were less than the predicted 12.4 LoF mutants, but this deficit was not sufficient to yield a significant pLI. Because the relatively short coding sequences of *CCN* genes limits interpretation of LoF mutants, exome data from a larger population will be needed confirm or exclude intolerance to LoF for CCN members other than *CCN1*. We also examined the frequency of missense mutations in the CCN family and found the largest deficits in *CCN1* and *CCN2* (Kaur and Roberts 2021), but again data from additional individuals would be required to confirm significance.

## *Thbs1*, a nonessential gene in mice that is intolerant to LoF in humans

Several matricellular genes that are not essential for viability in mice had significant pLI values in the gnomAD data, including *THBS1*, *THBS2*, *SPARC*, *SPOCK1*, and *TNR* (Kaur and Roberts 2021). *Thbs1*^*−/−*^ mice are viable, fertile, and appeared healthy except for lung inflammation (Lawler et al. 1998). Because *Thbs1* belongs to a family of 5 genes, essential roles for *Thbs1* could be masked by compensatory induction of other members in its gene family in *Thbs1*^*−/−*^ mice. However, such compensation is unlikely because double and triple knockout combinations with *Thbs2*, *Thbs3*, and *Comp* inactivation were also viable (Agah et al. 2002; Posey et al. 2008), In addition to compensation by paralogous genes, gene essentiality is quantitatively limited by cellular evolvability, wherein adaptive evolution activates compensatory mechanisms to bypass loss of an essential gene function (Liu et al. 2015).

In contrast to the *Thbs1*^*−/−*^ mouse data, significant deficits in the rates of missense and predicted LoF nonsense mutations in *THBS1* were found in the human gnomAD dataset (v2.1.1). Compared to the 56 expected LoF mutants, only 7 were observed, and the pLI for *THBS1* was 1.00, indicating this gene to be highly loss-intolerant (Kaur and Roberts 2021). In addition, none of the 7 individuals with *THBS1* LoF mutant alleles were homozygotes. The original report that mice with homozygous LoF mutants in *Thbs1* are viable and fertile excluded essential roles for this gene in embryonic development or reproduction (Lawler et al. 1998). Subsequent studies identified a role for *Thbs1* in ovarian follicular development and ovulation in mice and primates (McGray et al. 2011; Bender et al. 2019), but the limited effect on fertility is unlikely to account for the high pLI in humans. *THBS1* also had a significant deficiency in missense mutations (516 observed versus 721 expected, Z score = 2.72) (Kaur and Roberts 2021). However, the distribution of missense mutations in *THBS1* did not clearly identify specific residues or regions of the protein that mediate an essential function, apart from variants previously identified through genome-wide association studies linking a polymorphism in *THBS1* with early myocardial infarction and altered calcium binding (Topol et al. 2001; Carlson et al. 2008).

## Insights into protective functions of Thbs1 in mice

Aside from critical roles in embryonic development and adult reproduction, selective pressures against gene LoF can arise from any function that decreases the probability that an individual will survive long enough to successfully reproduce. Several relevant functions for matricellular proteins in postnatal survival have been revealed when adult knockout mice were subjected to specific stresses (Roberts et al. 2012; Calabro et al. 2014; Murphy-Ullrich and Sage 2014; Soto-Pantoja et al. 2015; Kim et al. 2018; Stenina-Adognravi and Plow 2019).

The diverse biological functions of thrombospondin-1 in regulation of angiogenesis, vascular homeostasis, connective tissue organization, responses to injury, synaptogenesis, and immune responses are mediated by its interactions with multiple receptors and extracellular ligands (Murphy-Ullrich 2019; Kaur et al. 2021). Thus, studies using knockout mice can also be informative to identify which receptors or ligands mediate specific functions. CD36 and CD47 are cell surface receptors for thrombospondin-1. the resistance of *Thbs1*^*−/−*^ mice to injuries caused by ischemia is phenocopied by *Cd47*^*−/−*^ but not by *Cd36*^*−/−*^ mice (Isenberg et al. 2007b). Correspondingly, LoF mutants in human *CD36* are relatively common (Yanai et al. 2000), whereas *CD47* was highly intolerant to LoF in the gnomAD data (pLI = 0.92) (Kaur and Roberts 2021), Additional receptors and interaction partners of thrombospondin-1 that share its elevated pLI in humans include stromal interaction molecule (*STIM1*, pLI = 0.78), loss of which is perinatal lethal in mice (Varga-Szabo et al. 2008), and low density lipoprotein receptor-related protein 1 (LRP1, pLI = 1.0), loss of which is embryonic lethal in mice (Herz et al. 1992). However, interaction partners other than thrombospondin-1 may also contribute to the essential functions of these receptors.

**Fig. 1:**
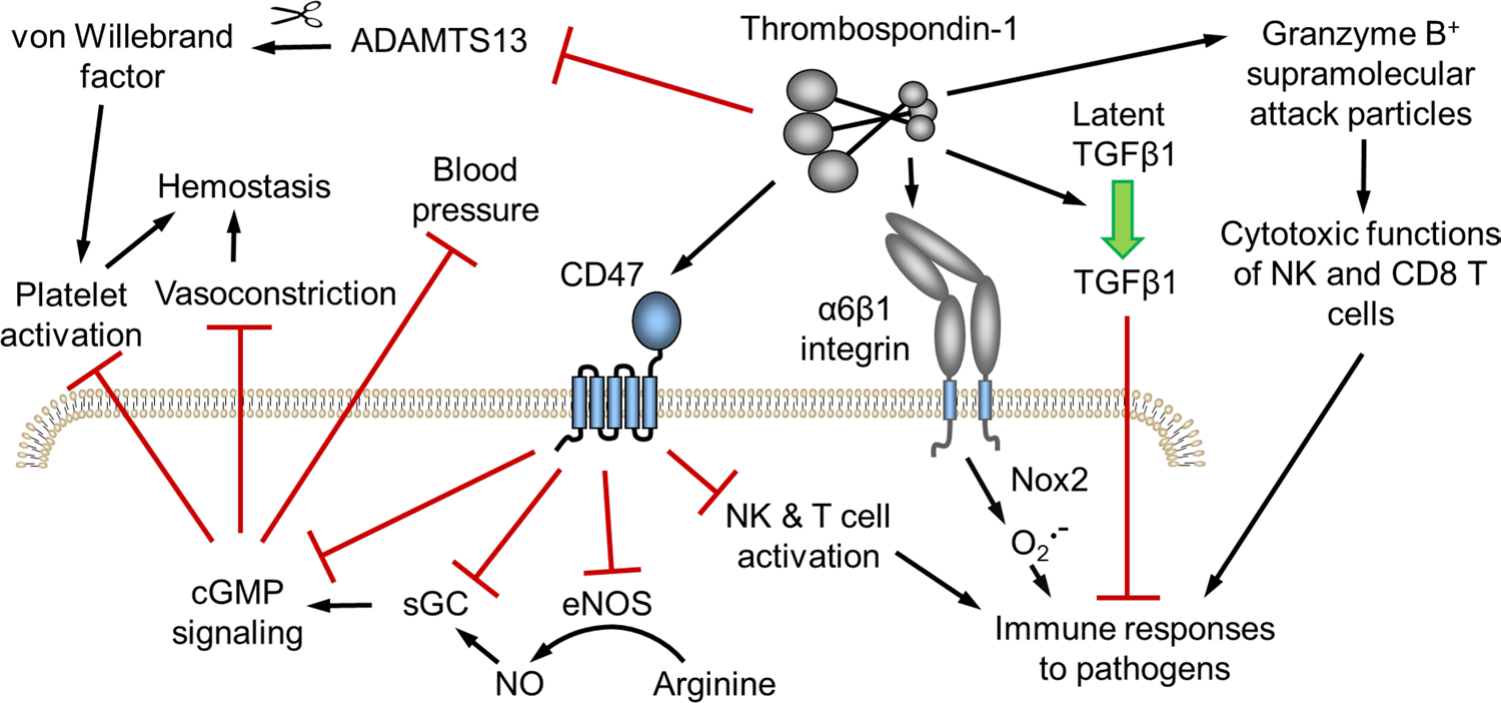
Thrombospondin-1 functions that may select against LoF mutants in humans. Hemostasis and maintenance of central blood pressure are essential for survival of acute injuries. Both processes are regulated by CD47-dependent thrombospondin-1 signaling, which inhibits the biosynthesis of nitric oxide (NO) by endothelial nitric oxide synthase (eNOS), the activation of soluble guanylate cyclase (sGC) by NO, and the downstream signaling mediated by cGMP-dependent protein kinases in vascular smooth muscle cells and platelets. Thrombospondin-1 also enhances platelet function by preventing the proteolytic inactivation of von Willebrand factor by ADAMTS13. Immune defenses against viral, bacterial, and fungal pathogens are also critical for survival to reproductive age. Thrombospondin-1 regulates innate and adaptive immune responses through CD47, interaction with α6β1 integrin that activates superoxide (O_2_^−^) production by NADPH oxidase-2 (Nox2), direct activation of immunosuppressive transforming growth factor-β1 (TGFβ1), and incorporation into supramolecular attack particles that mediate sustained delivery of granzyme B and perforin by NK and CD8 T cells to kill infected cells

Studies subjecting *Thbs1*^*−/−*^ mice and *Cd47*^*−/−*^ mice to defined stresses have identified several protective functions of CD47-dependent thrombospondin-1 signaling that could improve postnatal survival. These functions broadly involve enhancing the ability of the cardiovascular system to respond to acute injuries or supporting the immune system in responding to infectious diseases (Fig. 1).

Because thrombospondin-1 is a major component of platelet α-granules that is rapidly released at sites of injury, a potential role in hemostasis was one of the first hypotheses tested using *Thbs1*^*−/−*^ mice (Lawler et al. 1998). The initial examination of platelet function in *Thbs1*^*−/−*^ mice using washed platelets found no defect in platelet activation in vitro. However, a subsequent study revealed that a positive function of thrombospondin-1 in promoting platelet activation requires the presence of arginine, which was absent in the washed platelet medium. Arginine is the substrate for biosynthesis of nitric oxide, a potent negative regulator of platelet activation, and activation of *Thbs1*^*−/−*^ or *Cd47*^*−/−*^ platelets was defective when tested in the presence of physiological levels of nitric oxide or arginine (Isenberg et al. 2008b). Thrombospondin-1 signaling via CD47 redundantly inhibits nitric oxide biosynthesis and cGMP signaling in platelets and other vascular cells (Isenberg et al. 2006, 2008b) (Fig. 1). Thrombospondin-1 released from platelet α-granules thereby mediates positive feedback to overcome the physiological antithrombotic function of nitric oxide at sites of injury.

Additional studies of thrombospondin-1/CD47 signaling in vascular smooth muscle cells demonstrated that thrombospondin-1 enhances vasoconstriction and reduces local blood flow by blocking the function of nitric oxide to relax vascular smooth muscle (Isenberg et al. 2007a) (Fig. 1). Thus, signaling mediated by CD47 at an acute injury site where thrombospondin-1 is released by platelets can limit bleeding by simultaneously enhancing platelet hemostasis and vasoconstriction. These functions could cause individuals with LoF in *THBS1* or *CD47* to experience increased mortality due to blood loss when acutely injured.

Apart from these CD47-dependent signaling functions, thrombospondin-1 enhances hemostasis by its ability to directly inhibit ADAMTS13 (Bonnefoy et al. 2006) (Fig. 1). ADAMTS13 is a protease that cleaves von Willebrand factor, which inhibits the critical function of von Willebrand factor to mediate platelet activation and thrombus formation of when exposed to collagen at a site of vascular injury (Bonnefoy et al. 2006). ADAMTS13 was not significantly intolerant to LoF in humans (observed/expected LoF mutants = 0.52 (90% range 0.39–0.68), pLI = 0.0), but ADAMTS13 deficiency in mice can shorten their lifespan (Cassis et al. 2018). Another study identified a direct role of thrombospondin-1 to promote platelet adhesion and thrombus formation on exposed collagen by engaging its receptor CD36 (Kuijpers et al. 2014), which is not loss-intolerant in humans (Kaur and Roberts 2021). Thus, thrombospondin-1 released by platelets has multiple CD47-dependent and CD47-independent functions that limit bleeding at a site of injury.

Apart from its functions in hemostasis, regulation of nitric oxide signaling by thrombospondin-1/CD47 can improve survival of cardiovascular stresses that cause acute loss of blood pressure (Fig. 1). *Cd47*^*−/−*^ mice were more susceptible to death when subjected to isoflurane anesthesia using conditions that were well-tolerated by wild type mice (Isenberg et al. 2009). Death was caused by loss of blood pressure, and autonomic blockade similarly led to accelerated vascular collapse and death of *Thbs1*^*−/−*^ mice compared to wild type mice (Isenberg et al. 2009). The decreased stability of blood pressure regulation in the absence of thrombospondin-1 or CD47 could be another significant selective pressure that prevents accumulation of *THBS1* and *CD47* LoF mutants. Increased mortality could result from traumatic injuries or from blood loss as a complication of childbirth.

Unlike humans, laboratory mice live in an environment that minimizes their exposure to pathogens. Prior to the relatively recent development of antibiotics and vaccines, infectious disease was a major factor that limited survival of children to adulthood (Mackenbach and Looman 1988; DiLiberti and Jackson 1999). Thus, genes for which LoF significantly increases the risk of death from common infectious diseases may be loss-intolerant in humans without impairing the viability of laboratory mice. The functions of thrombospondin-1 in immune regulation are complex and are mediated by several thrombospondin-1 receptors (Forslow et al. 2007; Martin-Manso et al. 2008; Sarfati et al. 2008; Stein et al. 2016; Kaur et al. 2021). Correspondingly, loss of *Thbs1* in mice has been demonstrated to either increase or decrease their survival following exposure to different bacterial, viral, or fungal pathogens (Lawler et al. 1998; Martin-Manso et al. 2012a; Qu et al. 2018; Binsker et al. 2019; Arun et al. 2020).

Studies have identified several mechanisms by which thrombospondin-1 protects mice from specific pathogens. Initially, *Thbs1*^*−/−*^ mice were reported to have a chronic lung inflammatory phenotype (Lawler et al. 1998), which was subsequently attributed to loss of the ability of thrombospondin-1 to activate latent TGFβ1 (Crawford et al. 1998). The increased immunosuppressive activity of TGFβ1 in wild type mice would then prevent lung inflammation. This could also occur independent of infection, which is consistent with a report that thrombospondin-1 limits injury and collagen and CCN2 expression in lungs treated with bleomycin (Ezzie et al. 2011). However, the originally reported spontaneous lung inflammation in *Thbs1*^*−/−*^ mice may relate to exposure to a specific pathogen in the vivarium because the lung phenotype was lost when the mice were rederived in a different vivarium (Isenberg et al. 2008a). In another study, thrombospondin-1 protected mice from lung injury caused by *Pseudomonas aeruginosa* by inhibiting pathogen and host proteolytic activities (Qu et al. 2018).

Loss of *Thbs1* or *Cd47* in mice may also increase death from infectious diseases by impairing T cell and natural killer cell immunity (Fig. 1). CD47 is required for optimal defense against some bacterial, viral and *Candida albicans* fungal infections (Lindberg et al. 1996; Navarathna et al. 2015; Nath et al. 2018). Gene expression profiling indicated protective effects of thrombospondin-1 and CD47 on innate and adaptive immune cells (Navarathna et al. 2015; Nath et al. 2018). Thrombospondin-1 also enhances superoxide production via NADPH oxidase-2 in macrophages and neutrophils in an integrin α6β1-dependent manner (Martin-Manso et al. 2008; Roberts et al. 2017). The catalytic subunit of NADPH oxidase-2 encoded by *CYBB* is highly loss intolerant (observed/expected LoF mutants = 0.04 (0.01–0.19), pLI = 1.00) (Karczewski et al. 2020).

Recently, thrombospondin-1 was identified as an essential component of supramolecular attack particles produced by CD8 T cells and natural killer cells (Ambrose et al. 2020; Balint et al. 2020). Supramolecular attack particles are coated with a fragment of thrombospondin-1 and deliver the cytotoxic agents granzyme B and perforin to target cells, and the sustained delivery mediated by these particles increases that killing of refractory targets (Chang et al. 2022).

## Conclusions

Studies have identified beneficial as well as detrimental effects of *Thbs1* gene disruption on the ability of mice to survive exposure to specific pathogens or respond to a variety of physiological stresses (McMaken et al. 2011; Martin-Manso et al. 2012b; Soto-Pantoja et al. 2015; Zhao et al. 2015; Qu et al. 2018; Arun et al. 2020). Although each of the mechanisms outlined in this review have the potential to contribute to the selective pressure preventing loss of function in *THBS1* in humans, it is premature to rank their relevance. To date, the evidence is most compelling for protective functions of thrombospondin-1 in hemostasis to account for its loss-intolerance. Future genome wide association studies may identify additional missense mutations in *THBS1* that contribute to essential functions in humans and provide clues to the specific thrombospondin-1 interaction partners involved. The insights gained from stress models that revealed protective functions of *Thbs1* and *Cd47* in mice may also be applicable for identifying molecular mechanisms underlying the deficits in LoF and missense mutants observed for other thrombospondins and members of other matricellular gene families in humans.

## Data Availability

All data is contained in the manuscript or the indicated public databases.

## Abbreviations

CCN: Cellular communication network
gnomAD: Genome aggregation database
LoF: Loss of function
pLI: Probability of loss-of-function intolerance
THBS: Thrombospondin
